# Angular Tuning Properties of Low Threshold Mechanoreceptors in Isolated Rat Whisker Hair Follicles

**DOI:** 10.1523/ENEURO.0175-22.2022

**Published:** 2022-11-24

**Authors:** Akihiro Yamada, Hidemasa Furue, Jianguo G. Gu

**Affiliations:** 1 Department of Anesthesiology and Perioperative Medicine, University of Alabama at Birmingham, Birmingham, AL 35294; 2 Department of Neurophysiology, Hyogo Medical University, Nishinomiya 663-8501, Japan

**Keywords:** angular tuning, directional selectivity, low threshold mechanoreceptors, sensory encoding, tactile, whisker hair follicle

## Abstract

Angular tuning is preferential sensory response to a directional stimulus and is observed in the whisker tactile system. In whisker hair follicles, there are at least three types of low threshold mechanoreceptors (LTMRs): rapidly adapting (RA), slowly adapting type 1 (SA1), and slowly adapting type 2 (SA2). These LTMRs display angular tuning but their properties remain incompletely studied. Here, we used isolated rat whisker hair follicles and pressure-clamped single-fiber recordings to study angular tuning of these LTMRs. Angular tuning was determined with impulses elicited by ramp-and-hold deflection of whisker hair in 24 directions each at 15° for a total of 360°. We show that RA display impulses during ramp-up, both ramp-up and ramp-down, or ramp-down dynamic phases. Both SA1 and SA2 respond to angular stimuli with slowly adapting impulses in most angles. However, SA1 and SA2 show rapidly adapting responses in other angles. All the three types of LTMRs display strong angular tuning, and there is no significant difference in angular tuning index among them. Population wise, the majority of SA1 are tuned in the caudal direction, a large part of SA2 is tuned in the rostral direction, and RAs are tuned in multiple directions. In the angles showing strong tuning, the three LTMRs respond to increased stimulation amplitudes with increased impulse numbers in a hyperbola relationship, and the responsiveness based on impulse numbers is SA2 > SA1 > RA. Our findings provide new information on angular tuning properties of LTMRs in whisker hair follicles and help to understand directional encoding.

## Significance Statement

Angular tuning in the whisker tactile system is essential in life of rodents. Here, we studied angular tuning of three types of low threshold mechanoreceptors (LTMRs) in whisker hair follicles: rapidly adapting (RA), slowly adapting type 1 (SA1), and slowly adapting type 2 (SA2). All three types of LTMRs display strong angular tuning in response to whisker hair deflection. Population wise, SA1 are largely tuned to the caudal direction, SA2 are tuned mainly to the rostral direction, and RA are tuned to multiple directions. The three LTMRs respond to increased whisker hair deflection amplitudes with increased impulse numbers, and the responsiveness is SA2 > SA1 > RA. Our findings provide new insights into directional encoding by whisker hair follicle LTMRs.

## Introduction

The whisker tactile system, from whisker hair follicles in the periphery to the barrel cortex in the brain, is essential for environmental exploration, tactile discrimination, and social interaction in rodents. The rodent whisker tactile system is an important model system in neuroscience for addressing questions such as how neuronal activity encode sensory stimuli, and how the activity of sensory neurons in turn is “read out” in the brain to give rise to sensory perception and behavioral responses (Adibi, 2019). Rodents can perform tactile discrimination such as differentiating the texture and shape of an object by repetitively sweeping their whisker hairs around the object in forward (protraction) and backward (retraction) directions (Adibi, 2019). Touching an object with whisker hairs generates mechanic force within whisker hair follicles. This leads to activation of low threshold mechanoreceptors (LTMRs) and initiates sensory impulses at the terminals of Aβ-afferent nerves in whisker hair follicles (Leiser and Moxon, 2007). The nerve impulses at Aβ-afferent terminals encode tactile information such as magnitude, velocity, frequency, and direction of tactile stimuli, which are then conveyed to the whisker tactile system in the CNS (Lichtenstein et al., 1990; Minnery et al., 2003; Lee and Simons, 2004; Furuta et al., 2020).

Several morphologically distinct types of LTMRs such as Merkel discs, lanceolate endings, and reticular endings (also known as Ruffini-like endings) have been identified within whisker hair follicles (Halata and Munger, 1980; Takahashi-Iwanaga, 2000; Ebara et al., 2002). Functionally, LTMRs in whisker hair follicles can be classified into rapidly adapting (RA), slowly adapting type1 (SA1), and slowly adapting type 2 (SA2) LTMRs based on their responses to ramp-and-hold deflection of whicker hairs (Gottschaldt et al., 1973; Sonekatsu and Gu, 2019). RA LTMRs only fire impulses during the ramp (dynamic) phase of hair deflection and do not fire any impulse during the holding (static) phase of hair deflection (Tonomura et al., 2015; Sonekatsu and Gu, 2019). In contrast, SA1 LTMRs and SA2 LTMRs respond to ramp-and-hold hair deflection in both dynamic and static phase. SA1 LTMRs and SA2 LTMRs fire impulses in an irregular manner and a regular manner, respectively (Wellnitz et al., 2010). It has been suggested that ring sinus Merkel discs, reticular or Ruffini-like endings, and lanceolate endings in whisker hair follicles are functionally SA1, SA2 ,and RA LTMRs, respectively (Halata and Munger, 1980; Ebara et al., 2002; Abraira and Ginty, 2013; Sonekatsu and Gu, 2019; Furuta et al., 2020) . Molecular mechanisms underlying tactile transduction at Merkel discs have recently been uncovered with Piezo2 channels being identified as mechanical transducers located on Merkel cells and their associated Aβ-afferent terminals (Ikeda et al., 2014; Ranade et al., 2014; Woo et al., 2014).

Previous *in vivo* studies in rats and mice have shown that RA LTMRs and SA LTMRs display directional selectivity, or angular tuning (Lichtenstein et al., 1990; Shoykhet et al., 2000; Kwegyir-Afful et al., 2008; Furuta et al., 2020). Angular tuning has been observed in neurons along the whisker tactile pathways including the trigeminal ganglion, brainstem, thalamus, and barrel cortex (Lichtenstein et al., 1990; Minnery et al., 2003; Lee and Simons, 2004; Hemelt et al., 2010). Angular tuning is normally defined as responses to directional deflections of principal whisker hairs that evoke the largest response magnitude. Angular tuning is also observed in mouse body hair follicle lanceolate endings which are RA LTMRs derived from Aδ-afferent endings (Rutlin et al., 2014). The angular tuning of RA LTMRs in body hair follicles is largely attributable to the directional location of mechanoreceptors within the body hair follicles (Rutlin et al., 2014). Directional location of mechanoreceptors within whisker hair follicles also is suggested to attribute to angular tuning of SA1 LTMRs in rat whisker hair follicles (Furuta et al., 2020). However, angular tuning of RA LTMRs in rat whisker hair follicles does not appear to be related to locations of their mechanoreceptors (Furuta et al., 2020).

Previous studies on angular tuning of LTMRs in whisker hair follicles are performed *in vivo* in anesthetized rats and mice using extracellular recordings made from trigeminal ganglion neurons or intra-axon recordings made from infraorbital nerves (Lichtenstein et al., 1990; Kwegyir-Afful et al., 2008; Furuta et al., 2020). Angular tuning measured *in vivo* is affected by several factors, including intrinsic angular tuning properties of LTMRs within whisker hair follicles as well as tissue mechanics (or stiffness) of the skin that surrounds whisker hair follicles, and the muscle as well as connective tissues that attached to whisker hair follicles (Bush et al., 2016). In addition, anesthesia used in *in vivo* studies may affect impulse activity of LTMRs and thereby affecting the measurement of angular tuning. In the present study, to investigate the intrinsic angular tuning properties of LTMRs without the influence of the tissues surrounding whicker hair follicles, we used isolated whisker hair follicles and pressure-clamped single-fiber recording technique.

## Materials and Methods

### *Ex vivo* whisker hair follicle preparations

Sprague Dawley rats of both male and female weighing 300–500 g were used for making *ex vivo* whisker hair follicle preparations. Animal care and use conformed to NIH *Guidelines for Care and Use of Experimental Animals*. Experimental protocols were approved by the Institutional Animal Care and Use Committee (IACUC) at the University of Alabama at Birmingham. In brief, animals were anesthetized with 5% isoflurane and then killed by decapitation. Whisker pads were dissected out from orofacial areas of rats by using a pair of dissecting scissors and placed in a 35-mm Petri dish that contained Krebs bath solution (see below). Under a dissection microscope, fat tissues that covered whisker hair follicles were removed by using a pair of fine tweezers to expose individual whisker hair follicles. Whisker hair follicles located at the central region of the whisker pad including B2, B3, C2, C3, D2, D3, and D4 whisker hair follicles together with their whisker afferent bundles and hair shafts were then gently pulled out from both right and left whisker pads. The capsule of each whisker hair follicle was cut open at the end part of the capsule to facilitate solution exchange in the whisker hair follicle. Each whisker hair follicle was affixed on the sylgard-coated bottom of a recording chamber with tissue pins. The end of the whisker afferent bundle was sharply cut with a surgical knife and the nerve bundle was then affixed to the bottom of the recording chamber by a U-shaped tissue anchor. The *ex vivo* whisker hair follicle preparation was submerged in Krebs bath solution contained (in mm): 117 NaCl, 3.5 KCl, 2.5 CaCl_2_, 1.2 MgCl_2_, 1.2 NaH_2_PO_4_, 25 NaHCO_3_, and 11 glucose. The pH of the Krebs bath solution was adjusted to 7.3 with HCl or NaOH and osmolarity adjusted to 325 mOsm with sucrose. The Krebs solution was saturated with 95% O_2_ and 5% CO_2_ during experiments.

### Pressure-clamped single-fiber recordings from whisker afferent never fibers

The pressure-clamped single-fiber recording was performed to measure impulses in response to whisker hair deflection in the same manner described in our previous studies (Sonekatsu and Gu, 2019; Sonekatsu et al., 2020). In brief, the recording chamber was mounted on the stage of an Olympus BX50WI microscope. The whisker hair follicle preparation was briefly exposed to a mixture of 0.1% dispase II plus 0.1% collagenase in the Krebs bath solution for 1 min, and the enzymes were then quickly washed off with the normal Krebs bath solution. This gentle enzyme treatment was to help separating individual afferent fibers so that a single fiber could be aspirated into the recording electrode and pressure-clamped for single fiber recordings. Recording electrodes for pressure-clamped single-fiber recordings were made by thin-walled borosilicate glass tubing without filament (inner diameter 1.12 mm, outer diameter 1.5 mm, World Precision Instruments). They were fabricated by using P-97 Flaming/Brown Micropipette Puller (Sutter Instrument Co) and the tip of each electrode was fire polished by a microforge (MF-900, Narishige) and final tip size was 10–12 μm in diameter. The recording electrode was filled with Krebs bath solution, mounted onto an electrode holder which was connected to a high-speed pressure-clamp (HSPC) device (ALA Scientific Instruments) for fine controls of intraelectrode pressures. Under a 40× objective, the end of a single whisker afferent nerve was first separated from whisker afferent nerve bundle by applying a low positive pressure (∼10 mmHg or 0.19 Psi) from the recording electrode. The end of the single nerve fiber was then aspirated into the recording electrode by a negative pressure at approximately −10 mmHg. Once the end of the nerve fiber entered into the recording electrode in ∼10 μm, the electrode pressure was readjusted to −3 ± 2 mmHg and maintained at the same pressure throughout the experiment. Nerve impulses evoked by whisker hair deflection (see below) and conducted along a single whisker afferent fiber were recorded under the I_0_ configuration and amplified using a Multiclamp 700B amplifier (Molecular Devices). Electrical signals were amplified 100–1000 times and sampled at 25 kHz with AC filter at 0.1 Hz and Bessel filter at 3 kHz under AC membrane mode (Digidata 1440A, Molecular Devices). All experiments were performed at the room temperature of 23–24°C.

### Angular stimulation of whisker hairs

Whisker hair was trimmed to have the length of ∼8 mm. A mechanical probe, made with an L-shaped glass pipette, was mounted on a pipette holder and controlled by a programmable manipulator (MPC-325, Sutter). The trimmed hair shaft was fit into the end of the mechanical probe, and the position of the whisker hair shaft was adjusted to neutral position at which the hair shaft was not bended toward any direction. To produce angular deflection of the whisker hair, the mechanical probe was moved in a ramp-and-hold manner in 24 directions at the increment of 15° each direction (360° total). The interval between any two angular deflections was 5 s. Unless otherwise specified, the ramp-and-hold deflection consisted of a 234-ms ramp-up to 200 μm (dynamic phase), 4.75-s hold at 200 μm (static phase), and a 234-ms ramp-down to the original position. All angular deflections were made by a programmable manipulator that was controlled by a software (Multi-Link, Sutter), and the probe movement as well as nerve impulses were simultaneously recorded using the pClamp 10 software.

In the present study, whisker hair follicles at the central region of both right and left whisker pads were used. The angular deflection used the afferent root of each whisker hair follicle as a reference point, which is ∼45° in dorsal-caudal direction in the head axes *in vivo* as was determined anatomically. Angular deflection of each whisker hair in our recording chamber started first in the direction same as its afferent root’s direction, i.e., 45° in dorsal-caudal direction in the coordinate of head axes. In each experiment, disregard using a left or a right whisker hair follicle, the angular deflection was applied clockwise in 15° increment. The conversion from the afferent-root-referenced coordinate to the *in vivo* head axes was as follow. For a left-side whisker hair follicle, 45°, 135°, 225°, and 315° clockwise whisker hair deflections in reference to the afferent root were equivalent to *in vivo* whisker hair deflection in caudal, ventral, rostral and dorsal, directions, respectively. For a right-side whisker hair follicle, 45°, 135°, 225°, and 315° clockwise whisker hair deflections in reference to the afferent root were equivalent to *in vivo* whisker hair deflection in dorsal, rostral, ventral, and caudal directions, respectively. All other angles could be converted to the head axes in the same manner.

### Data analysis

Data were collected from whisker hair follicles of three male and 22 female animals. We observed no signs that there were differences between the male and female rats, thus their data were considered together for data analysis. Impulses recorded from single whisker afferent fibers were analyzed using the Clampfit 10 software. Unless otherwise specified, impulses induced during the dynamic phase (234 ms) and static phase (4.75 s) of angular whisker hair deflection were combined together to represent angular response. Impulse numbers or frequency were used to represent angular response of LTMRs to whisker hair deflection. SA1 and SA2 LTMRs were defined by the coefficient of variance (CV) of interimpulse intervals, with CV value ≥ 0.5 being considered as SA1 LTMRs and CV < 0.5 as SA2 LTMRs, based on previous studies on the regularity of SA LTMRs in mouse hair skin (Wellnitz et al., 2010) and whisker hair follicles (Sonekatsu and Gu, 2019). In the cases when CV values were at the borderline of 0.5, we also used impulse frequency to aid for classifying SA1 (lower frequency) or SA2 (higher frequency; Sonekatsu and Gu, 2019). Sensitive angles were defined as the angles at which the angular responses (impulse numbers) were ≥80% of maximum angular response. Tuning direction was the vector of angular response. Four general tuning directions were defined, caudal (rostral-to-caudal ±45°), ventral (dorsal-to-ventral ±45°), rostral (caudal-to-rostral ±45°), and dorsal (ventral-to-dorsal ±45°) direction. The angle size of high sensitivity was defined as the size of the angle at which responses were ≥80% of maximal impulses. Tuning index was calculated and used as a measure of how strong angular tuning was for individual LTMRs (Taylor and Vaney, 2002). The tuning index (D) was calculated as D = Σvi/Σri (i.e., D = vector sum/scalar sum), where vi are vector magnitudes pointing in the direction of the stimulus and having length, ri, equal to the number of impulses recorded during that stimulus (Bellavance et al., 2010). D can range from 0, when the responses are equal in all stimulus directions, to 1, when a response is obtained only for a single stimulus direction (Bellavance et al., 2010). Curve fitting for the relationship between whisker hair deflection amplitude and LTMR response were made with a hyperbola equation Y = (Ymax*X)/(Kd+X), where Y is the response (impulse numbers), X is whisker hair deflection amplitude, and Kd is deflection amplitude at which 50% of maximal response is produced. All data analyses were performed using GraphPad Prism (version 8). Unless otherwise indicated, all data were reported as mean ± SEM of *n* independent observations. Statistical significance was evaluated using the Kruskal–Wallis (nonparametric) test with Dunn’s *post hoc* tests for multiple group comparison, Mann–Whitney (nonparametric) test or Student’s *t* tests for two group comparison. Differences were considered to be significant with *p < 0.05, **p < 0.01, ***p < 0.001, and not significant (ns) with *p* ≥ 0.05.

## Results

### Angular response of rapidly adapting (RA) LTMRs

**Figure 1.**
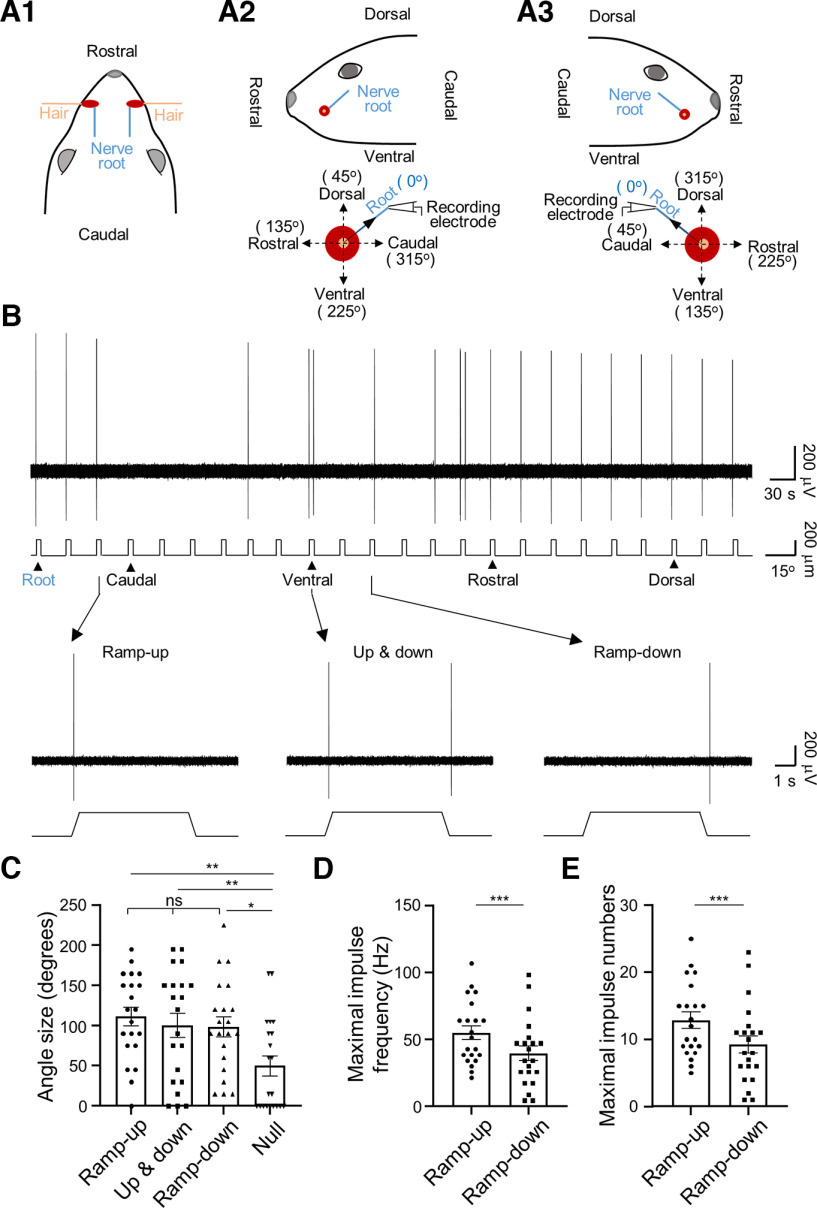
Angular responses of RA LTMRs in whisker hair follicles of rats. ***A1–A3***, Positions of a left and a right whisker hair (orange), their follicles (red) and afferent roots (blue) in head axes viewed from top of the head (***A1***), left side (***A2***, top) and right side (***A3***, top). In the diagrams, only one hair follicle with its afferent root on each side is shown for clarity. For either the left (***A2***, top panel) or the right (***A3***, top panel) whisker hair follicle, its afferent root tilts ∼45° toward dorsal-caudal direction. The positions described here are applicable for the whisker hair follicles located at the central region of whisker pads. ***A2***, bottom, ***A3***, bottom, *In vitro* recording of afferent impulses while whisker hair is angularly deflected. In experiments, for either a left-side or a right-side hair follicle, the first hair deflection was always applied in the same direction as its afferent root’s direction (solid black arrow indicated, 45° dorsal-caudal direction). Whisker hair deflections were subsequently applied clockwise in a 15° increment. ***B***, Sample trace (top panel) shows RA LTMR impulses in response to ramp-and-hold whisker hair deflection in 24 angular directions each at the amplitude of 200 μm. The recording was from a left C2 whisker hair follicle. The scale bar to the right indicates the angle size of whisker hair deflection. The 24 directions of whisker hair deflection started with 1st deflection in afferent root direction. Bottom three traces are impulses at the expanded time scale from arrow-indicated angles in the top panel, one angle induced responses only in the ramp-up phase (left), another angle induced impulses in both the ramp-up and ramp-down phases (middle), and the third angle induced impulses only in the ramp-down phase (right). In all 24 directions, no impulses were evoked during the static phase. ***C***, Summary data (*n* = 21) of the sizes of the angles showing impulses only in the ramp-up phase, impulses in both the ramp-up and ramp-down phases, impulses only in the ramp-down phase, and the sizes of angles showing no response (null angle). ***D***, ***E***, Summary data (*n* = 21) of RA LTMR impulse frequency (***D***) and number (***E***) in the ramp-up (circles) and ramp-down (squares) phases at the most sensitive angles. Data represent mean ± SEM, ***p < 0.001.

We used *ex vivo* rat whisker hair follicle preparation and applied pressure-clamped single-fiber recording technique to study responses of LTMRs to angular deflection of rat whisker hairs (Fig. 1*A1–A3*). In each recording, the afferent root of each whisker hair follicle was used as a reference point so that angular deflection of a whisker hair in our recording chamber can be converted to the deflection directions in head axes *in vivo* (Fig. 1*A1–A3*). As shown in Figure 1*A1–A3*, for whisker hair follicles at the central region of whisker pads, afferent root of these whisker hair follicles tilted ∼45° toward dorsal-caudal direction (Fig. 1*A2*,*A3*). In experiments, for either a left-side or a right-side hair follicle, the first hair deflection was applied in the same direction as its afferent root’s direction, i.e., 45° dorsal-caudal direction. Subsequent whisker hair deflections were applied clockwise in a 15° increment (Fig. 1*A2*,*A3*). Angular responses were measured as whisker afferent impulses in response to ramp-and-hold deflection of a whisker hair in 24 different directions for a total of 360° (Fig. 1*B*).

In one type of LTMR response, angular deflection in each direction evoked impulses only during the dynamic phase. Using an angular whisker hair deflection of 200 μm, impulses could be elicited in many angles and phases, including during ramp-up, both ramp-up and ramp-down, or ramp-down (Fig. 1*B*). In some angles, deflection of whisker hairs failed to elicit any impulse (null angle). Regardless of direction, no impulse was elicited during the static phase of the ramp-and-hold whisker hair deflection. Thus, these were RA LTMRs. The average sizes of angles for which RA LTMRs responded only in ramp-up phase were 111 ± 12° (*n* = 21), in both ramp-up and ramp-down phases were 100 ± 15° (*n* = 21), and only in ramp-down dynamic phase was 99 ± 13° (*n* = 21; Fig. 1*C*), with no significant difference observed. The sizes of the angles showing no response or null angle was 50 ± 12° (*n* = 21; Fig. 1*C*), and was significantly narrower than those with responses (*p* < 0.05). Average maximal RA impulse frequency was 54.9 ± 5.2 Hz (*n* = 21; Fig. 1*D*) for the angular responses occurring during ramp-up phase, significantly higher than that during ramp-down phase (39.5 ± 5.6 Hz, *n* = 21, *p* < 0.001; Fig. 1*D*). Average maximal impulse count was 12.9 ± 1.2 (*n* = 21; Fig. 1*E*) for the angular responses occurring during ramp-up phase, significantly more than that during ramp-down phase (9.2 ± 1.3, *n* = 21, *p* < 0.001; Fig. 1*E*).

**Figure 2.**
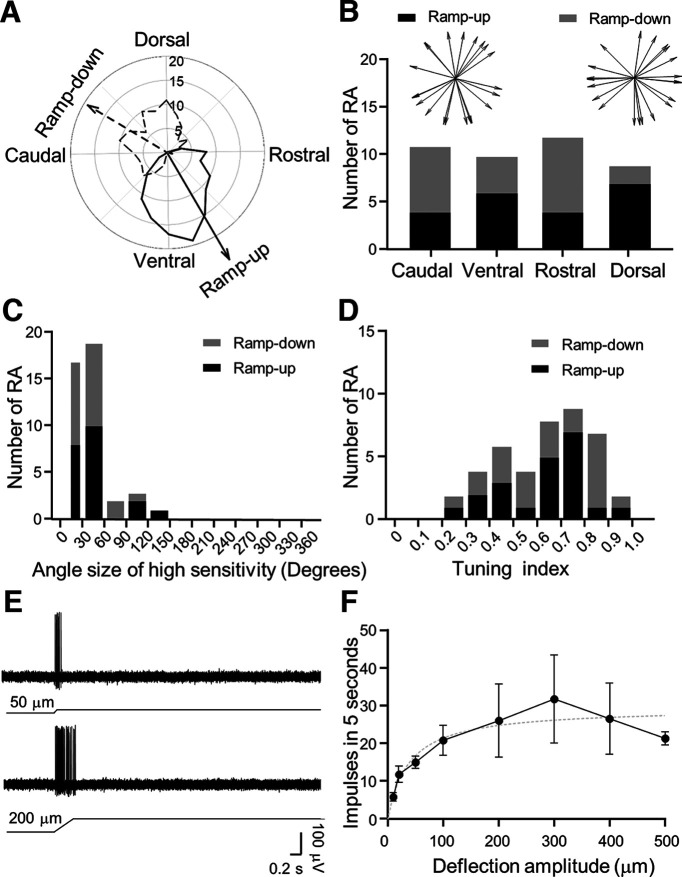
Angular tuning parameters and amplitude encoding of RA LTMRs. ***A***, Polar plot shows an example of angular responses of an RA LTMR. Solid and dashed black lines indicate angular responses in the ramp-up and ramp-down phases, respectively. Solid black arrow and dashed black arrow indicate vectors of the ramp-up response and ramp-down response, respectively. Concentric circles and numbers indicate impulse numbers. ***B***, Bar graph shows numbers of RA LTMRs displaying angular tuning in the caudal (caudal ±45°), ventral (ventral ±45°), rostral (rostral ±45°), and dorsal (dorsal ±45°) directions. Black bars, ramp-up responses; gray bars, ramp-down responses. Inset, Tuning direction of each RA LTMR (*n* = 21). ***C***, Histogram shows the number of RA LTMRs with the angle sizes of high sensitivity. The angle size of high sensitivity was defined as the angle size at which responses were ≥80% of maximal impulses. The bin is 30°. Black bars, ramp-up responses; gray bars, ramp-down responses. ***D***, Histogram shows the number of RA LTMRs with different tuning indexes. The bin of tuning index is 0.1. Black bars, ramp-up responses; gray bars, ramp-down responses. ***E***, Sample traces of RA LTMR impulses induced by a 50-μm (top) and a 200-μm (bottom) ramp-and-hold deflection of a whisker hair in the most sensitive direction. ***F***, RA impulse numbers induced by whisker hair deflection at the most sensitive angle at the amplitudes of 10, 20, 50, 100, 200, 300, 400, and 500 μm (*n* = 5 for 10–200 μm, 4 for 300–400 μm, and 3 for 500 μm). Dotted line is the curve fitting the experimental data. Data represent mean ± SEM.

To quantitatively determine tuning properties of RA LTMRs, impulses evoked by 200-μm whisker hair deflection at each angle were plotted in the polar coordinate system (polar plot), and tuning direction was then calculated as a vector of the overall impulses (Fig. 2*A*,*B*). The data were grouped into four general angular categories (Fig. 2*A*), caudal direction (caudal ±45°), dorsal direction (dorsal ±45°), rostral direction (rostral ±45°), and ventral direction (ventral ±45°). RA LTMRs showed strong angular tuning, and the ramp-up and ramp-down responses usually were tuned in opposite directions (Fig. 2*A*). More RA LTMRs showed dorsal and ventral angular tuning in the ramp-up phase, and caudal and rostral angular tuning in the ramp-down phase (Fig. 2*B*). However, population wise, RA LTMRs were tuned to multiple directions rather than to a dominant direction (Fig. 2*B*). The angle sizes of high sensitivity, defined as the sizes of angles at which impulse count was ≥80% maximal angular response, ranged from angle sizes of 15–150° for RA LTMRs, and most had narrow angles with the sizes from 15° to 60° (Fig. 2*C*). Overall, the angle sizes of high sensitivity for RA LTMRs were 36 ± 7° (*n* = 21). Tuning index has been commonly used to describe how strong angular tuning is in response to angular stimulation, with the tuning index value of 0 being no angular tuning and 1 being the strongest tuning. Tuning index was determined for each RA LTMR in response to angular stimulation. For ramp-up responses, the tuning index was in the range of 0.2–1 in all 21 recordings, but the majority of recordings had a tuning index of 0.6–0.8 (Fig. 2*D*). For ramp-down responses, the tuning index was also in the range of 0.2–1 in all 21 recordings, but the majority of recordings had a tuning index of 0.8–0.9 (Fig. 2*D*). Overall, the tuning index was 0.626 ± 0.041 (*n* = 21) for ramp-up responses and 0.640 ± 0.045 (*n* = 21) for ramp-down responses (Fig. 2*D*). These results indicate that individual RA LTMRs mostly had strong angular tuning.

At the most sensitive tuning direction where maximal angular response was elicited, we quantitatively measured the relationship between whisker hair deflection amplitudes and RA LTMR responses in rat whisker hair follicles (Fig. 2*E*,*F*). We tested whisker hair deflections ranging from 10 μm to 500 μm. RA impulses showed progressive increases in response to whisker hair deflections from 10 to 300 μm, and the response appeared to be plateaued subsequently. The deflection amplitude-response relationship could be fit with a hyperbola equation Y = (Ymax*X)/(Kd+X), where Y is response (impulse numbers), X is deflection amplitude, and Kd is deflection amplitude at which 50% of maximal response is produced (Fig. 2*F*). Overall, Kd value was 25 ± 11 μm (*n* = 3–5; Fig. 2*F*) for the hyperbola relationship between whisker hair deflection amplitude and RA LTMR response.

### Angular response of slowly adapting type 1 (SA1) LTMRs

**Figure 3.**
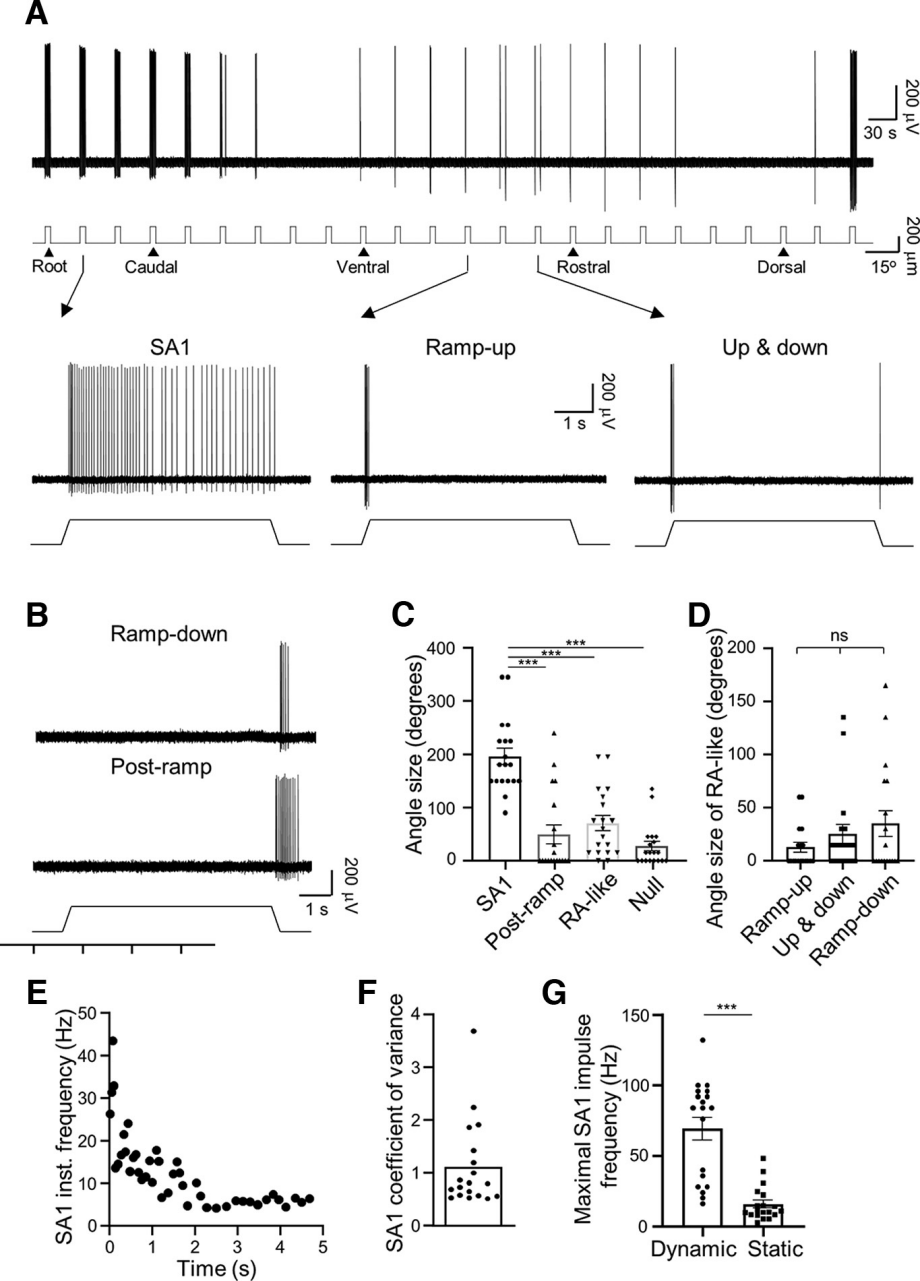
Angular responses of SA1 LTMRs in whisker hair follicles. ***A***, Sample trace (top panel) shows SA1 LTMR impulses in response to ramp-and-hold whisker hair deflection in 24 angular directions each at the amplitude of 200 μm. Whisker hair deflection at each angle is indicated under the sample trace. Bottom three traces are impulses at the expanded time scale from arrow-indicated angles in the top panel, one was the most sensitive angle (−30° to the caudal direction) at which maximal numbers of impulses were evoked in both the dynamic and static phase (left panel), another was the angle at which impulses were evoked only in the ramp-up dynamic phase (middle panel), and the third was the angle at which impulses were evoked in both ramp-up and ramp-down dynamic phases (right panel). The sample traces were recorded from a left C3 whisker hair follicle. ***B***, A different SA1 displaying impulses during the ramp-down dynamic phase (top panel) or impulses during and after ramp-down dynamic phase (bottom trace, postramp). The sample traces were recorded from a left C3 whisker hair follicle. ***C***, Summary data (*n* = 19) of the sizes of the angles showing impulses in both dynamic and static phase (SA1), during and after ramp-down phase (postramp), only during dynamic phase (RA-like), and the sizes of the angles showing no response (null angle). ***D***, Summary data (*n* = 19) of the angle sizes of RA-like subclasses with impulses only during ramp-up phase, impulses in both the ramp-up and ramp-down phases, impulses only during the ramp-down phase. ***E***, Instantaneous frequency of impulses at the most sensitive angle shown in ***A***. ***F***, Summary data (*n* = 19 recordings) of coefficient of variance of interevent intervals of impulses at the most sensitive angles exemplified in ***A***. ***G***, Summary data (*n* = 19) of impulse frequency of SA1 LTMRs in the dynamic phase (234 ms) and static phase (4.75 s) at most sensitive angles exemplified in ***A***. Data represent mean ± SEM, *p < 0.05, **p < 0.01, ***p < 0.001, ns, not significantly different.

In the second type of LTMR responses, an angular deflection of 200-μm evoked impulses during both the dynamic and static phase for many angles (Fig. 3*A*). The impulses displayed high irregularity in interimpulse intervals (see Fig. 3*E*,*F*) and thereby were SA1 responses (Wellnitz et al., 2010). However, for other angles the whisker hair deflection evoked impulses only during the dynamic phases of ramp-up, ramp-up and ramp-down (up and down), or ramp-down (Fig. 3*A*,*B*), and collectively we termed these impulses RA-like responses. In addition, in some angles, impulses occurred during and after ramp-down phase (Fig. 3*B*), which were termed postramp responses in the present study. There were also angles at which whisker hair deflections failed to elicit any impulses (null angle; Fig. 3*A*). For all SA1 LTMRs tested with whisker hair deflection, the sizes of angles that elicited impulses in both the dynamic and static phase (SA1 angle) were 198 ± 16° (*n* = 19), which were significantly greater than the sizes of angles that elicited impulses only in the dynamic phase (RA-like angle, 73 ± 14°, *n* = 19, *p* < 0.001; Fig. 3*C*). SA1 angle sizes were also significantly larger than that the sizes of the postramp impulse angles (52 ± 18°, *n* = 19, *p* < 0.001; Fig. 3*C*) and the null angles (27 ± 9°, *n* = 19, *p* < 0.001; Fig. 3*C*). For RA-like responses, the sizes of angles that elicited impulses in ramp-up only, ramp-up and ramp-down, and ramp-down only were 13 ± 5° (*n* = 19), 25 ± 9° (*n* = 19), and 35 ± 12° (*n* = 19), respectively, and no significant difference was observed (Fig. 3*D*).

At the most sensitive angles where impulses occurred in both the dynamic and static phase of whisker hair deflection, the instantaneous frequency calculated from interimpulse intervals was highly irregular (Fig. 3*E*). The coefficient of variance of interimpulse intervals was 1.11 ± 0.19 (*n* = 19 recordings; Fig. 3*F*), indicating high irregularity of interimpulse intervals. The result of irregular impulses indicated these were SA1 LTMRs. For whisker hair deflection of 200 μm in the most sensitive angle, impulse frequency was 69.3 ± 8.0 Hz (*n* = 19) in the dynamic phase, significantly higher than that in the static phase (15.6 ± 2.8 Hz, *n* = 19, *p* < 0.001; Fig. 3*G*).

**Figure 4.**
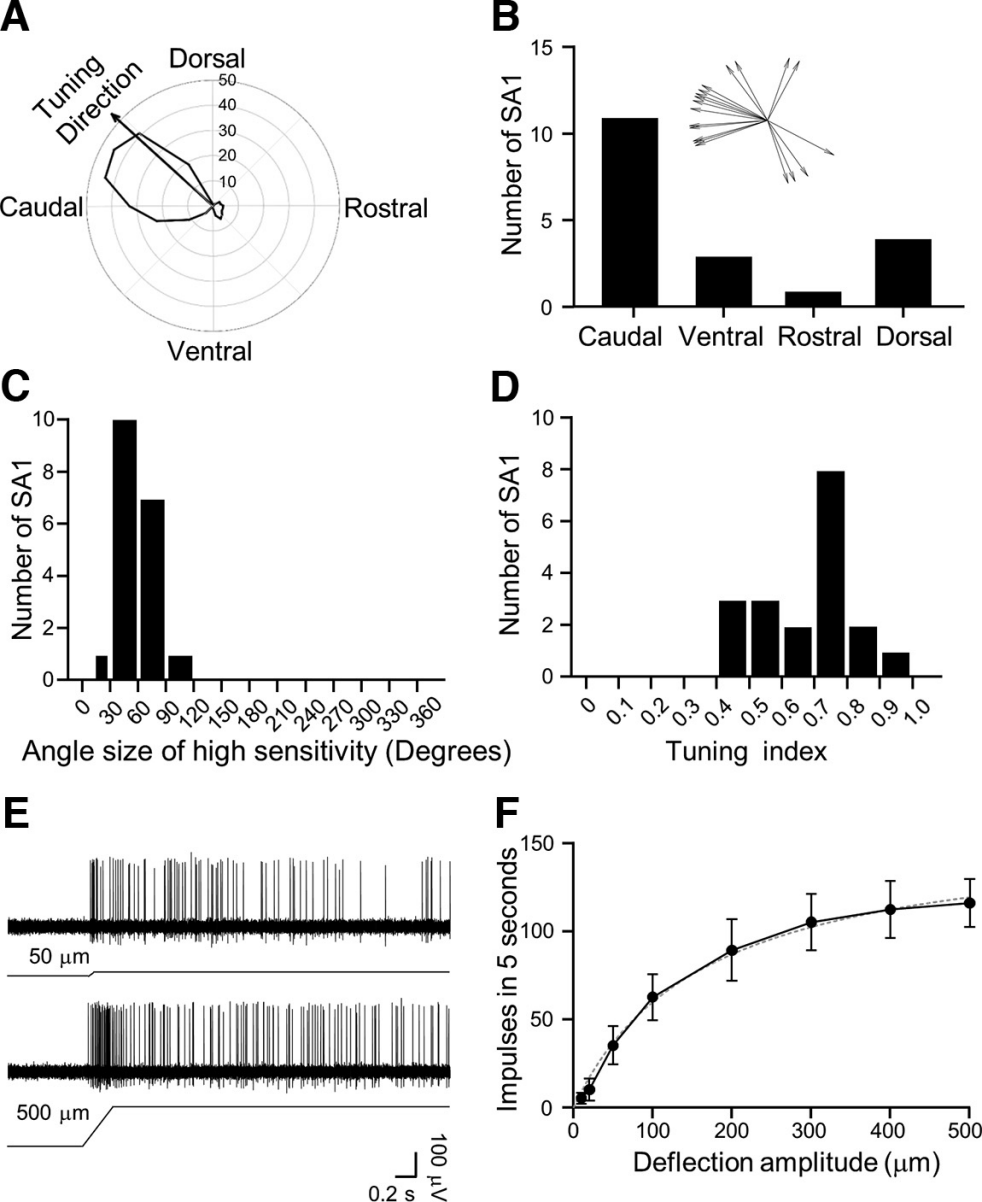
Angular tuning parameters and amplitude encoding of SA1 LTMRs. ***A***, Polar plot shows an example of angular response of an SA1 LTMR. Arrow in the plot indicates the vector of the angular response. Concentric circles and numbers indicate impulse numbers. ***B***, Bar graph shows the number of SA1 LTMRs displaying angular tuning to the caudal (caudal ±45°), ventral (ventral ±45°), rostral (rostral ±45°), and dorsal (dorsal ±45°) directions. Inset, Tuning directions of each SA1 LTMR recorded (*n* = 19). ***C***, Histogram shows distribution of SA1 LTMRs with the angle sizes of high sensitivity. The bin is 30°. ***D***, Histogram shows distribution of SA1 LTMRs with different tuning indexes. The bin of tuning index is 0.1. ***E***, Sample traces show SA1 impulses induced by a 50-μm (top) and a 500-μm (bottom) ramp-and-hold deflection of a whisker hair in the most sensitive direction. ***F***, SA1 impulse numbers induced by whisker deflection at the amplitudes of 10, 20, 50, 100, 200, 300, 400, and 500 μm (*n* = 4). Dotted line is the curve fitting the experimental data. Data represent mean ± SEM.

To quantitatively determine tuning properties of SA1 LTMRs, impulses evoked by 200-μm whisker hair deflection at each angle were plotted in the polar coordinate system, and tuning direction was then calculated as a vector of the overall impulses (Fig. 4*A*,*B*). Of a total 19 SA1 LTMRs recorded, 11/19 (57.9%) were tuned to the caudal direction, 4/19 (21.1%) to the dorsal direction, 3/19 (15.8%) to the ventral direction, and 1/19 (5.3%) to the rostral direction (Fig. 4*B*). Thus, the caudal direction was the most common tuning direction for SA1 LTMRs in rat whisker hair follicles (Fig. 4*A*,*B*). We determined the angle sizes of high sensitivity for each SA1 LTMR recorded. Of 19 SA1 LTMRs recorded, 17/19 (89.5%) had the angle sizes of high sensitivity between 30° and 90° (Fig. 4*C*). Overall, the average angle size of high sensitivity of these SA1 LTMRs was 52.1 ± 4.5° (Fig. 4*C*, *n* = 19). Tuning index was determined for SA1 LTMRs. Tuning index ranged from 0.4 to 1 in all 19 recordings, but a large fraction of recordings (8/19) had tuning index from 0.7 to 0.8 (Fig. 4*D*), suggesting many SA1 LTMRs were highly tuned angularly. Overall, tuning index was 0.68 ± 0.03 (*n* = 19; Fig. 4*D*), indicating a strong angular tuning of SA1 LTMRs in rat whisker hair follicles.

Having determined the angular tuning, we further quantitatively measured, at the most sensitive tuning direction, the relationship between whisker hair deflection amplitude and response of SA1 LTMRs. The deflection amplitudes from 10 to 500 μm were tested. SA1 impulses showed a progressive increase in impulse numbers with increased deflection amplitudes up to 300 μm and nearly reached to a plateau level afterward (Fig. 4*E*,*F*, *n* = 4). The deflection amplitude-response relationship could be best fit into the hyperbola equation with a Kd value of 105 ± 34 μm (*n* = 4).

### Angular response of slowly adapting type 2 (SA2) LTMRs

**Figure 5.**
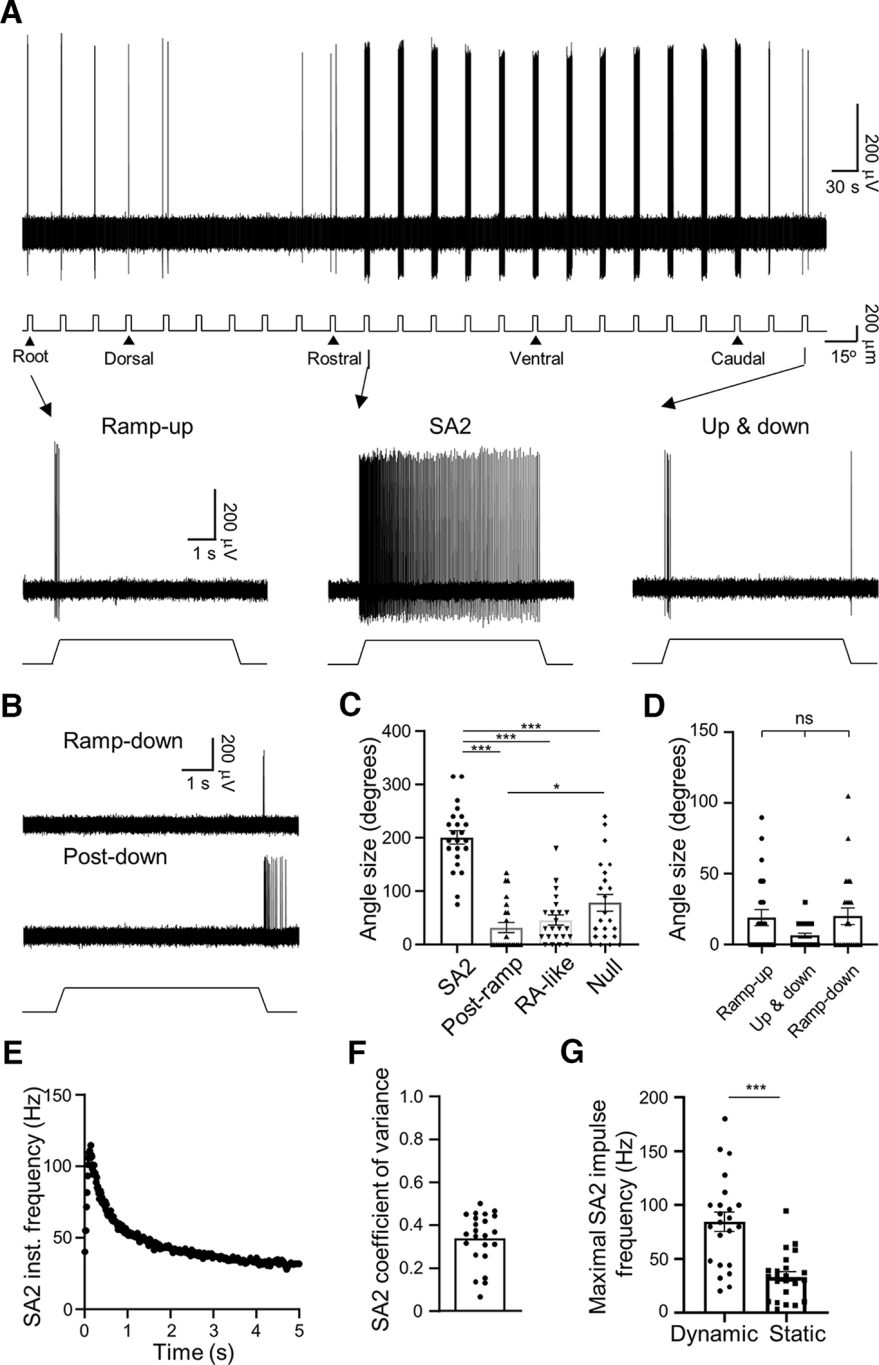
Angular responses of SA2 LTMRs in whisker hair follicles. ***A***, Sample trace (top panel) shows SA2 LTMR impulses in response to ramp-and-hold whisker hair deflection in 24 angular directions each at the amplitude of 200 μm. Whisker hair deflection at each angle is indicated under the sample trace. Bottom three traces are impulses at the expanded time scale from arrow-indicated angles in the top panel, one was the angle at which impulses were evoked only in the ramp-up dynamic phase (left panel), another was the most sensitive angle at which maximal numbers of impulses were evoked in both the dynamic and static phase (middle panel), and the third was the angle at which impulses were evoked in both the ramp-up and ramp-down dynamic phases (right panel). The sample traces were recorded from a right D3 whisker hair follicle. ***B***, A different SA2 displaying impulses during the ramp-down dynamic phase (top panel) or impulses during and after ramp-down dynamic phase (bottom trace, postramp). The sample traces were recorded from a right D3 whisker hair follicle. ***C***, Summary data (*n* = 23) of the sizes of the angles showing impulses in both dynamic and static phase (SA2), during and after ramp-down phase (postramp), only during dynamic phase (RA-like), and the sizes of the angles showing no response (null angle). ***D***, Summary data (*n* = 23) of the angle sizes of RA-like subclasses with impulses only during ramp-up phase, impulses in both the ramp-up and ramp-down phases, impulses only in the ramp-down phase. ***E***, Instantaneous frequency of impulses at the most sensitive angle shown in ***A***. ***F***, Summary data (*n* = 23) of coefficient of variance of interevent intervals of impulses at the most sensitive angles exemplified in ***A***. ***G***, Summary data (*n* = 23) of impulse frequency of SA2 LTMRs in the dynamic phase (234 ms) and static phase (4.75 s) at most sensitive angles exemplified in ***A***. Data represent mean ± SEM, *p < 0.05, ***p < 0.001, ns, not significantly different.

In the third type of LTMR response, 200-μm angular deflection of whisker hairs evoked impulses during both the dynamic and static phases for many angles (Fig. 5*A*). The impulses displayed high regularity in interimpulse intervals (see Fig. 5*E*,*F*) and thereby were SA2 responses (Wellnitz et al., 2010). However, for some angles whisker hair deflection evoked impulses only during the ramp-up, ramp-up and ramp-down (up and down), or ramp-down dynamic phase (RA-like; Fig. 5*A*,*B*). In addition, in some angles, impulses occurred during and after ramp-down phase (postramp; Fig. 5*B*). There were also angles at which whisker hair deflections failed to elicit any impulses (null angle; Fig. 5*A*). For all SA2 LTMRs tested with whisker hair deflection, the sizes of angles that elicited impulses in both the dynamic and static phases (SA2 angle) were 200 ± 13° (*n* = 23), which was significantly greater than the sizes of RA-like angles that elicited impulses in dynamic phase only (46 ± 9°, *n* = 23, *p* < 0.001; Fig. 5*C*). The sizes of SA2 angles were also significantly larger than the sizes of angles of the postramp impulses (31 ± 10°, *n* = 23, *p* < 0.001) and the null angles (78 ± 16°, *n* = 23, *p* < 0.001; Fig. 5*C*). For RA-like responses, the sizes of the angles that elicited impulses in ramp-up only, ramp-up and ramp-down, and ramp-down only were 19 ± 6° (*n* = 23), 7 ± 2° (*n* = 23), and 20 ± 6° (*n* = 23), respectively, and no significant difference was observed (Fig. 5*D*).

At the highest sensitive angles, the instantaneous frequency calculated from the interimpulse intervals showed high regularity (Fig. 5*E*). The coefficient of variance of interevent interval of impulses elicited at the highest sensitive angles was 0.32 ± 0.02 (*n* = 23), indicating high regularity of the impulses (Fig. 5*F*). The result of highly regular impulses indicated these were SA2 LTMRs. For whisker hair deflection of 200 μm at the most sensitive angles, impulse frequency was 84.2 ± 8.8 Hz (*n* = 23) in the dynamic phase, significantly higher than that in the static phase (33.2 ± 4.6 Hz, *n* = 23, *p* < 0.001; Fig. 5*G*).

**Figure 6.**
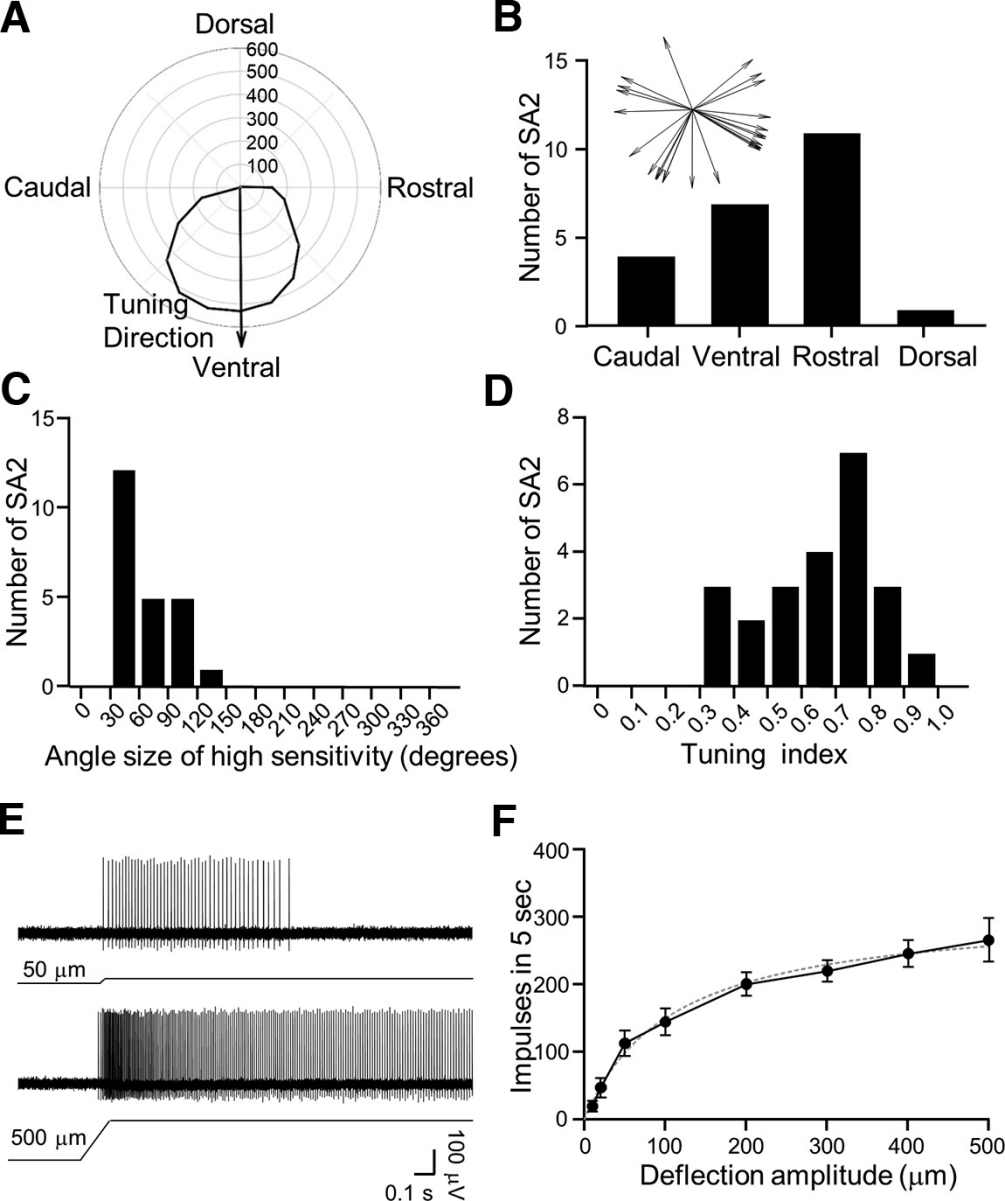
Angular tuning parameters and amplitude encoding of SA2 LTMRs. ***A***, Polar plot shows an example of angular response of an SA2 LTMR. Solid arrow indicates the vector of angular responses. Concentric circles and numbers indicate impulse numbers. ***B***, Bar graph shows numbers of SA2 LTMRs that display angular tuning in the caudal (caudal ±45°), ventral (ventral ±45°), rostral (rostral ±45°), and dorsal (dorsal ±45°) directions. Inset is the tuning direction of each SA2 LTMR recorded (*n* = 23). ***C***, Histogram shows distribution of SA2 LTMRs with the angle sizes of high sensitivity. The bin is 30°. ***D***, Histogram shows distribution of SA2 LTMRs with different tuning index. The bin of tuning index is 0.1. ***E***, Sample traces of SA2 impulses induced by a 50-μm (top) and a 500-μm (bottom) ramp-and-hold deflection of a whisker hair in the most sensitive direction. ***F***, SA2 impulse numbers induced by whisker hair deflection at the amplitudes of 10, 20, 50, 100, 200, 300, 400, and 500 μm (*n* = 6). Dotted line is the curve fitting the experimental data. Data represent mean ± SEM.

We determined tuning direction of SA2 LTMRs using the polar plot for the impulses evoked by 200-μm whisker hair deflection (Fig. 6*A*,*B*). Of a total 23 SA2 LTMRs recorded, 11/23 (47.8%) SA2 were tuned to the rostral direction, 7/23 (30.4%) to the ventral direction, 4/23 (17.4%) to the caudal direction, and 1/23 (4.3%) to the dorsal direction (Fig. 6*B*). Thus, the rostral direction was the most common and dorsal direction the least common tuning direction for SA2 LTMRs in rat whisker hair follicles (Fig. 6*A*,*B*).

We determined the angle sizes of high sensitivity of SA2 LTMRs recorded from whisker afferent fibers. Of 23 SA2 LTMRs recorded, the angle of high sensitivity was in the size range from 30° to 150°, and >50% SA2 LTMRs recorded showed the angle size of high sensitivity in 30–60° (Fig. 6*C*). Overall, the average angle size of high sensitivity of these SA2 LTMRs were 60.0 ± 5.9° (*n* = 23; Fig. 6*C*). Tuning index was determined for each SA2 LTMR recorded with angular stimulation. Tuning index was in the range of 0.3–1 in all 23 recordings, and the majority of recordings had tuning index of 0.7–0.8 (Fig. 6*D*). Overall, tuning index was 0.659 ± 0.035 (*n* = 23; Fig. 6*D*), indicating a strong angular tuning in SA2 LTMRs in rat whisker hair follicles. At the most sensitive tuning direction, we quantitatively measured the relationship between whisker hair deflection amplitude and SA2 response in rat whisker hair follicles (Fig. 6*E*,*F*, *n* = 6). We tested deflection amplitudes ranging from 10 to 500 μm. SA2 impulses showed a progressive increase in a manner that could be described by the hyperbola equation with a Kd value of 167 ± 48 μm (*n* = 6; Fig. 6*F*).

### Comparison of angular tuning of SA1, SA2, and RA LTMRs in whisker hair follicles

**Figure 7.**
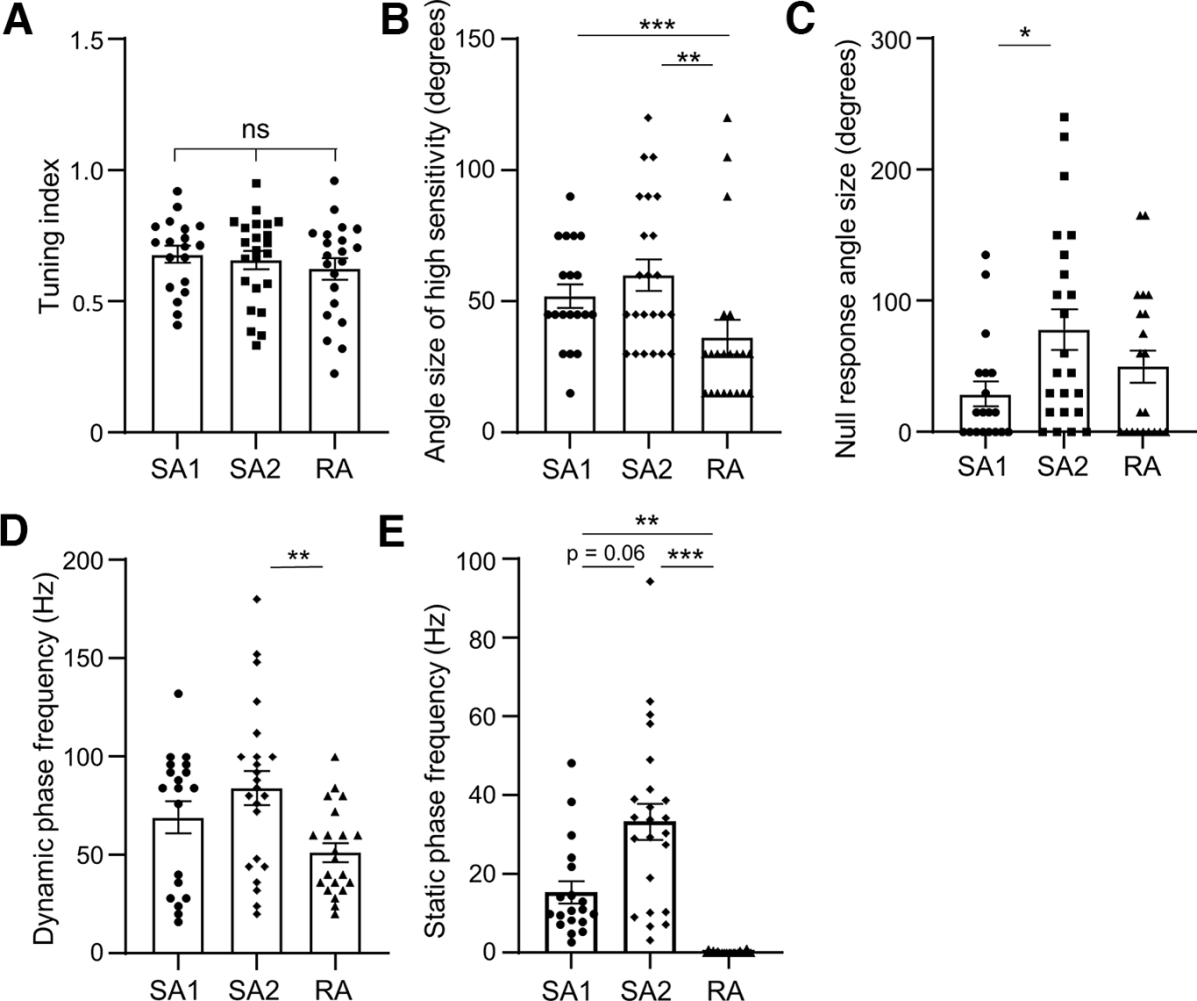
Comparison of angular tuning and amplitude encoding among RA, SA1, and SA2 LTMRs. ***A***, Comparison of tuning index among SA1 (*n* = 19), SA2 (*n* = 23), and RA (*n* = 21). ***B***, Comparison of the angle sizes of high sensitivity among SA1 (*n* = 19), SA2 (*n* = 23), and RA (*n* = 21). ***C***, Comparison of angle sizes of null responses among SA1 (*n* = 19), SA2 (*n* = 23), and RA (*n* = 21). ***D***, Comparison of impulse frequencies at the dynamic phase among SA1 (*n* = 19), SA2 (*n* = 23), and RA (*n* = 21). ***E***, Comparison of the impulse frequencies at the static phase among SA1 (*n* = 19), SA2 (*n* = 23), and RA (*n* = 21). From ***A–E***, impulses were elicited by whisker hair deflection at the amplitude of 200 μm in testing directions. Data represent mean ± SEM, *p < 0.05, **p < 0.01, ***p < 0.001, ns, not significantly different.

Figure 7 compares and contrasts the angular sensitivities of the three different LTMR types already shown in Figures 1-6. Data were the responses of these LTMRs to angular deflection of whisker hairs at the amplitude of 200 μm. All three types of LTMRs showed strong angular tuning with high tuning index, and tuning indexes were not significantly different among the three types of LTMRs (Fig. 7*A*). While SA1 and SA2 LTMRs showed similar average angle sizes of high sensitivity, RA displayed significantly narrower angles of high sensitivity in comparison with the angles of high sensitivity of both SA1 (*p* < 0.001; Fig. 7*B*) and SA2 LTMRs (*p* < 0.01; Fig. 7*B*). The angle sizes of null response, for which the angular stimulation did not evoke any impulses, were significantly narrower in SA1 LTMRs than that of SA2 LTMRs (*p* < 0.05; Fig. 7*C*). Determined at the most sensitive angles, the impulse frequency in dynamic phase was significantly lower in RA LTMRs (*n* = 21, *p* < 0.05) than in SA2 LTMRs (*n* = 23; Fig. 7*D*); the impulse frequency in the dynamic phase was not significantly different between SA1 LTMRs (*n* = 19), and SA2 LTMRs (*n* = 23; Fig. 7*D*). For the static phase, the impulse frequency of SA2 LTMRs (*n* = 23) was nearly significantly higher than that of SA1 LTMRs (*n* = 19, *p* < 0.06; Fig. 7*E*); RA LTMRs did not respond to angular whisker hair deflection (*n* = 21; Fig. 7*E*).

## Discussion

Using acutely isolated whisker hair follicles of rats and pressure-clamped single-fiber recording technique, the present study investigated responses of RA, SA1, and SA2 LTMRs in whisker hair follicles to directional stimuli to explore how these LTMRs encode directional tactile information. We show that all three LTMRs have strong angular tuning. Population wise, a large portion of SA1 LTMRs and SA2 LTMRs are tuned in the caudal-to-rostral and the rostral-to-caudal directions, respectively, and RA LTMRs are tuned to multiple directions without a dominant one. In the most sensitive direction, all three LTMRs encode amplitude of whisker hair deflection, and the responsiveness based on impulse numbers is SA2 > SA1 > RA. The present study provides new information on angular tuning of the three types of LTMRs in whisker hair follicles of rats.

There are several technical differences between the present work and several previous studies. First, the present study used isolated whisker hair follicles and performed *in vitro* recordings while previous studies performed *in vivo* recordings in anesthetized animals (Lichtenstein et al., 1990; Kwegyir-Afful et al., 2008; Furuta et al., 2020). The use of isolated whisker hair follicles allows us to study tuning properties of LTMRs within whisker hair follicles without potential complications because of mechanics of tissues that surround whisker hair follicles (Bush et al., 2016). Second, in the present study we tested 24 angular directions to map angular response at each direction. On the other hand, previous studies tested 4 or 8 directions (Lichtenstein et al., 1990; Kwegyir-Afful et al., 2008; Rutlin et al., 2014; Furuta et al., 2020). Our study with 24 angular directions allowed us to observe transitional changes in angular responses. For example, we show that, both SA1 and SA2 LTMRs display rapidly adapting responses in some stimulation angles. For RA LTMRs, we also observed transitional changes, from ramp-up response only, both ramp-up and ramp-down response, to ramp-down response only. To our knowledge, these transitional changes have not been characterized previously. We have observed impulses after the end of ramp-down phase (poststimulation responses) in some SA1 and SA2 LTMRs, which may be because the mechanical tension of these LTMRs did not return to the basal level immediately after the withdraw of the mechanical probe. The third difference between the present study and previous studies is that we performed recordings from nerve fibers that innervate individual whiskers being studied while previous recordings were performed in a blinded manner from trigeminal neurons (Lichtenstein et al., 1990; Kwegyir-Afful et al., 2008). In addition to the above differences, the deflection ramp speed and amplitude as well as holding period were also different between the present work and previous studies. For example, our ramp-and-hold stimulation is relatively slower in speed, smaller in amplitude, and longer in holding period in comparison with previous studies (Lichtenstein et al., 1990; Shoykhet et al., 2000).

Individually, each RA, SA1, or SA2 LTMR has strong angular tuning as shown in the present study. Based on calculated tuning index, angular tuning of these three types of LTMRs are equally well, and there is no significant difference in tuning index among the three types of LTMRs. However, we found that RA LTMRs had relatively narrower angles of high sensitivity in comparison with the angle sizes of SA1 and SA2 LTMRs, suggesting that RA LTMRs may be better angularly tuned than SA1 and SA2 LTMRs. SA2 had relatively broader null response angles in comparison with SA1 LTMRs, suggesting that SA2 may be better angularly tuned than SA1 LTMRs. In contrast, angular tuning was shown to be better in SA LTMRs than RA LTMRs in previous *in vivo* studies with recordings from TG neurons in anesthetized rats and mice showed better (Lichtenstein et al., 1990; Kwegyir-Afful et al., 2008). The discrepancy in the degree of angular tuning between SA and RA LTMRs may be because of the subclassification of SA LTMRs into SA1 LTMRs and SA2 LTMRs in the present study. We show that at the best tuning angle for each LTMR, response to whisker hair deflection increases with the increases of whisker hair deflection amplitudes. The responsiveness based on impulse numbers is SA2 > SA1 > RA. Our results are consistent with a previous study showing that SA encode stimulation amplitude better than that of RA LTMRs (Shoykhet et al., 2000).

We show, as a whole population, SA1 LTMRs are tuned in the rostral-to-caudal direction since a large portion of SA1 LTMRs have response vectors pointing to the caudal direction. In contrast, a relatively large portion of SA2 LTMRs are tuned in the caudal-to-rostral direction. Our results contradict previous *in vivo* studies which showed that individual SA LTMRs were tuned to different directions rather than preferentially to a certain direction (Lichtenstein et al., 1990; Kwegyir-Afful et al., 2008). It should be noted that the previous *in vivo* recordings did not subclassify SA LTMRs into SA1 and SA2 LTMRs (Lichtenstein et al., 1990; Shoykhet et al., 2000; Kwegyir-Afful et al., 2008), which may be a reason of having weaker angular tuning in previous studies. In a more recent *in vivo* study, strong angular tuning was also shown in individual SA1 LTMRs being recorded but the small number of recordings in that study could not allow for determining population wise if SA1 LTMRs have angular tuning to a certain direction (Furuta et al., 2020). Population wise, the lack of angular tuning for SA LTMRs in previous studies may be because of the opposite tuning directions of SA1 LTMRs and SA2 LTMRs since the previous studies did not subclassify SA into SA1 and SA2 (Lichtenstein et al., 1990; Kwegyir-Afful et al., 2008). In contrast to SA1 and SA2 LTMRs, RA LTMRs as a population display much weaker angular tuning in comparison with SA1 and SA2 LTMRs. This is evidenced by our finding that, overall, RA LTMRs are not overwhelmingly outnumbered in a given direction although individual RA LTMRs show strong angular tuning. The lack of strong angular tuning for RA LTMRs as a whole population is in sharp contrast with RA LTMRs in body hair follicles of mice which were shown to be tuned in the rostral-to-caudal direction (Rutlin et al., 2014).

The angular tuning of the LTMRs in whisker hair follicles raises a question as whether there are tactile blind spots in whisker tactile system. Tactile information and resulting behavioral response are most likely the results of population responses of LTMRs to tactile stimuli. Since RA LTMRs in whisker hair follicles as a whole population respond almost equally well to whisker deflection in every direction, there should be no tactile blind spot in rats in terms of using their RA LTMRs of whisker hair follicles to detect stimuli from different directions. Mechanisms underlying angular tuning of LTMRs in whisker hair follicles are not fully understood. It has been thought that uneven distribution of the terminals of LTMRs within whisker hair follicles may contribute to angular tuning of LTMRs (Furuta et al., 2020). Consistently, the caudal-to-rostral angular tuning of Aδ-afferent RA LTMRs in body hair follicles is attributed to directional expression of mechanoreceptors (Abraira and Ginty, 2013). More recently, it has also been shown that the angular tuning of SA1 LTMRs in whisker hair follicles is attributed to the directional location of SA1 afferent terminals in whisker hair follicles (Furuta et al., 2020). In addition to the location of mechanoreceptors, angular tuning in terms of the degree of sensitive and null angles may be partially attributed to the areas of LTMR terminal arborization. Furthermore, the viscoelastic properties of tissues that surround LTMR terminals may also contribute to the degree of sensitive and null angles (Bush et al., 2016). The viscoelastic properties of these tissues may also play a role in the transitional responses observed in all three types of LTMRs. The use of isolated whisker hair follicles and pressure-clamped single-fiber recordings as shown in the present study may provide a useful model to further study the underlying mechanisms of angular tuning of LTMRs within whisker hair follicles and to help understand neuronal encoding of directional tactile information.

## Synthesis

Reviewing Editor: Miriam Goodman, Stanford University Decisions are customarily a result of the Reviewing Editor and the peer reviewers coming together and discussing their recommendations until a consensus is reached. When revisions are invited, a fact-based synthesis statement explaining their decision and outlining what is needed to prepare a revision will be listed below. The following reviewer(s) agreed to reveal their identity: NONE.

The reviewers are satisfied with that the revisions clarify what is being measured and what is not being evaluated. Rev2 has a few additional suggestions for you to consider regarding terminology. I ask you to consider these suggestions in making final revisions, but making these changes is entirely optional.

## References

[B1] Abraira VE, Ginty DD (2013) The sensory neurons of touch. Neuron 79:618–639. 23972592 10.1016/j.neuron.2013.07.051 PMC3811145

[B2] Adibi M (2019) Whisker-mediated touch system in rodents: from neuron to behavior. Front Syst Neurosci 13:40. 31496942 10.3389/fnsys.2019.00040 PMC6712080

[B3] Bellavance MA, Demers M, Deschenes M (2010) Feedforward inhibition determines the angular tuning of vibrissal responses in the principal trigeminal nucleus. J Neurosci 30:1057–1063. 20089914 10.1523/JNEUROSCI.4805-09.2010 PMC6633092

[B4] Bush NE, Schroeder CL, Hobbs JA, Yang AE, Huet LA, Solla SA, Hartmann MJ (2016) Decoupling kinematics and mechanics reveals coding properties of trigeminal ganglion neurons in the rat vibrissal system. Elife 5:e13969. 10.7554/eLife.13969 27348221 PMC4999311

[B5] Ebara S, Kumamoto K, Matsuura T, Mazurkiewicz JE, Rice FL (2002) Similarities and differences in the innervation of mystacial vibrissal follicle-sinus complexes in the rat and cat: a confocal microscopic study. J Comp Neurol 449:103–119. 10.1002/cne.10277 12115682

[B6] Furuta T, Bush NE, Yang AE, Ebara S, Miyazaki N, Murata K, Hirai D, Shibata KI, Hartmann MJZ (2020) The cellular and mechanical basis for response characteristics of identified primary afferents in the rat vibrissal system. Curr Biol 30:815–826.e5. 32004452 10.1016/j.cub.2019.12.068 PMC10623402

[B7] Gottschaldt KM, Iggo A, Young DW (1973) Functional characteristics of mechanoreceptors in sinus hair follicles of the cat. J Physiol 235:287–315. 4763992 10.1113/jphysiol.1973.sp010388 PMC1350747

[B8] Halata Z, Munger BL (1980) Sensory nerve endings in rhesus monkey sinus hairs. J Comp Neurol 192:645–663. 7419748 10.1002/cne.901920403

[B9] Hemelt ME, Kwegyir-Afful EE, Bruno RM, Simons DJ, Keller A (2010) Consistency of angular tuning in the rat vibrissa system. J Neurophysiol 104:3105–3112. 20668277 10.1152/jn.00697.2009 PMC3007639

[B10] Ikeda R, Cha M, Ling J, Jia Z, Coyle D, Gu JG (2014) Merkel cells transduce and encode tactile stimuli to drive Abeta-afferent impulses. Cell 157:664–675. 24746027 10.1016/j.cell.2014.02.026 PMC4003503

[B11] Kwegyir-Afful EE, Marella S, Simons DJ (2008) Response properties of mouse trigeminal ganglion neurons. Somatosens Mot Res 25:209–221. 18989828 10.1080/08990220802467612 PMC2597100

[B12] Lee SH, Simons DJ (2004) Angular tuning and velocity sensitivity in different neuron classes within layer 4 of rat barrel cortex. J Neurophysiol 91:223–229. 14507984 10.1152/jn.00541.2003

[B13] Leiser SC, Moxon KA (2007) Responses of trigeminal ganglion neurons during natural whisking behaviors in the awake rat. Neuron 53:117–133. 17196535 10.1016/j.neuron.2006.10.036

[B14] Lichtenstein SH, Carvell GE, Simons DJ (1990) Responses of rat trigeminal ganglion neurons to movements of vibrissae in different directions. Somatosens Mot Res 7:47–65. 2330787 10.3109/08990229009144697

[B15] Minnery BS, Bruno RM, Simons DJ (2003) Response transformation and receptive-field synthesis in the lemniscal trigeminothalamic circuit. J Neurophysiol 90:1556–1570. 12724362 10.1152/jn.00111.2003

[B16] Ranade SS, Woo SH, Dubin AE, Moshourab RA, Wetzel C, Petrus M, Mathur J, Begay V, Coste B, Mainquist J, Wilson AJ, Francisco AG, Reddy K, Qiu Z, Wood JN, Lewin GR, Patapoutian A (2014) Piezo2 is the major transducer of mechanical forces for touch sensation in mice. Nature 516:121–125. 25471886 10.1038/nature13980 PMC4380172

[B17] Rutlin M, Ho CY, Abraira VE, Cassidy C, Bai L, Woodbury CJ, Ginty DD (2014) The cellular and molecular basis of direction selectivity of Adelta-LTMRs. Cell 159:1640–1651. 25525881 10.1016/j.cell.2014.11.038 PMC4297767

[B18] Shoykhet M, Doherty D, Simons DJ (2000) Coding of deflection velocity and amplitude by whisker primary afferent neurons: implications for higher level processing. Somatosens Mot Res 17:171–180. 10895887 10.1080/08990220050020580

[B19] Sonekatsu M, Gu JG (2019) Functional properties of mechanoreceptors in mouse whisker hair follicles determined by the pressure-clamped single-fiber recording technique. Neurosci Lett 707:134321. 31181301 10.1016/j.neulet.2019.134321

[B20] Sonekatsu M, Yamada H, Gu JG (2020) Pressure-clamped single-fiber recording technique: a new recording method for studying sensory receptors. Mol Pain 16:1744806920927852. 10.1177/1744806920927852 32420801 PMC7235654

[B21] Takahashi-Iwanaga H (2000) Three-dimensional microanatomy of longitudinal lanceolate endings in rat vibrissae. J Comp Neurol 426:259–269. 10.1002/1096-9861(20001016)426:2<259::AID-CNE7>3.0.CO;2-N 10982467

[B22] Taylor WR, Vaney DI (2002) Diverse synaptic mechanisms generate direction selectivity in the rabbit retina. J Neurosci 22:7712–7720. 12196594 10.1523/JNEUROSCI.22-17-07712.2002 PMC6757986

[B23] Tonomura S, Ebara S, Bagdasarian K, Uta D, Ahissar E, Meir I, Lampl I, Kuroda D, Furuta T, Furue H, Kumamoto K (2015) Structure-function correlations of rat trigeminal primary neurons: emphasis on club-like endings, a vibrissal mechanoreceptor. Proc Jpn Acad Ser B Phys Biol Sci 91:560–576. 26666306 10.2183/pjab.91.560 PMC4773582

[B24] Wellnitz SA, Lesniak DR, Gerling GJ, Lumpkin EA (2010) The regularity of sustained firing reveals two populations of slowly adapting touch receptors in mouse hairy skin. J Neurophysiol 103:3378–3388. 20393068 10.1152/jn.00810.2009 PMC2888253

[B25] Woo SH, Ranade S, Weyer AD, Dubin AE, Baba Y, Qiu Z, Petrus M, Miyamoto T, Reddy K, Lumpkin EA, Stucky CL, Patapoutian A (2014) Piezo2 is required for Merkel-cell mechanotransduction. Nature 509:622–626. 24717433 10.1038/nature13251 PMC4039622

